# *LDlinkR*: An R Package for Rapidly Calculating Linkage Disequilibrium Statistics in Diverse Populations

**DOI:** 10.3389/fgene.2020.00157

**Published:** 2020-02-28

**Authors:** Timothy A. Myers, Stephen J. Chanock, Mitchell J. Machiela

**Affiliations:** Division of Cancer Epidemiology and Genetics, National Cancer Institute, Rockville, MD, United States

**Keywords:** linkage disequilibrium, population genetics, bioinformatics, R, LDlink

## Abstract

Genomic research involving human genetics and evolutionary biology relies heavily on linkage disequilibrium (LD) to investigate population-specific genetic structure, functionally map regions of disease susceptibility and uncover evolutionary history. Interactive and powerful tools are needed to calculate population-specific LD estimates for integrative genomics research. LDlink is an interactive suite of web-based tools developed to query germline variants in 1000 Genomes Project population groups of interest and generate interactive tables and plots of LD estimates. As an expansion to this resource, we have developed an R package, *LDlinkR*, designed to rapidly calculate statistics for large lists of variants and LD attributes that eliminates the time needed to perform repetitive requests from the web-based LDlink tool. *LDlinkR* accelerates genomic research by providing efficient and user-friendly functions to programmatically interrogate and download pairwise LD estimates from expansive lists of genetic variants. *LDlinkR* is a free and publicly available R package that can be installed from the Comprehensive R Archive Network (CRAN) or downloaded from https://github.com/CBIIT/LDlinkR.

## Introduction

Linkage disequilibrium (LD) is a population-based parameter that describes the degree to which an allele of one genetic variant is inherited or correlated with an allele of a nearby genetic variant within a given population (Bush and Moore, 2012). Measures of LD are important for biomedical research and are useful in a wide range of applications. Population geneticists calculate LD to assess population structure and population history (Mueller, 2004) and LD analysis can be employed to detect natural selection and estimate allelic age (Slatkin, 2008). Genome-wide association studies (GWAS) also apply LD to identify genotype-phenotype associations for a range of disease phenotypes (Welter et al., 2013; Chanock et al., 2014). GWAS is carried out by either genotyping a small fraction of known variants to “tag” other highly correlated variants or with whole genome sequencing (WGS) data. However, once loci associated with a disease or trait are identified, the process of disentangling plausible functional variants that explain the observed signal requires careful assessment of local LD structure in the study population (Machiela and Chanock, 2015). Thus, knowledge of population-specific LD patterns and user-friendly tools to calculate LD measures are essential for biomedical research.

Here, we present *LDlinkR*, an R package which facilitates researchers in exploring LD structure in a native R environment. *LDlinkR* leverages the computing resources of the cloud by harnessing the storage capacity and processing power of the LDlink web server to calculate computationally expensive LD statistics. This eliminates the need to store large VCF files and data sets locally as well as frees up local computing resources. Also, rather than requiring repeated completion of web-based forms as in our web-based LDlink tool (Machiela and Chanock, 2015), *LDlinkR* enables researchers familiar with the R statistical programing language (R Core Team, 2018) to rapidly calculate LD statistics for expansive lists of genetic variants and easily integrate results into local analytic pipelines for future statistical analyses. *LDlinkR* accelerates population genetics research by providing a fluid workflow for calculating LD metrics from diverse ancestral populations using the R environment.

## Implementation

**FIGURE 1:**
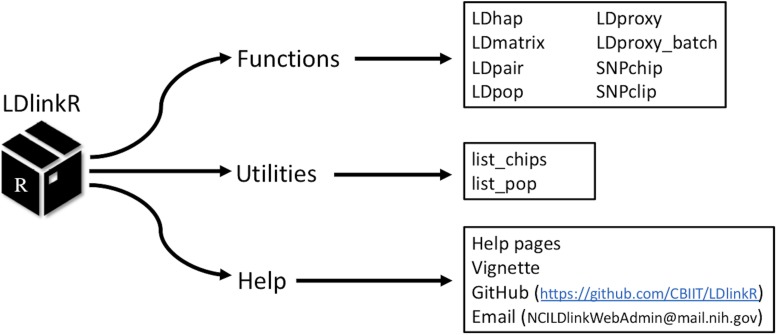
Structure of the *LDlinkR* package.

LDlink^1^ is a popular web-based bioinformatic tool for assessing LD across human population groups (Machiela and Chanock, 2015). *LDlinkR* has many of the same user input requirements as the classic LDlink tool; however, *LDlinkR* introduces advanced features and allows for flexible computation of large queries not possible using the LDlink web interface. Reference haplotype data for *LDlinkR* originates from the publicly released Phase 3 (Version 5) data from the 1000 Genomes (1000G) Project (1000 Genomes Project Consortium, 2012). The release contains 2,504 individuals (i.e., over 5,000 haplotypes) spanning 26 ancestral population groups. Phased haplotype information is available from continental populations (e.g., European, African, and Admixed American) and sub-populations (e.g., Finnish, Gambian, and Peruvian). Any combination of super or sub-population is permitted as input for *LDlinkR* queries. The second required input for *LDlinkR* modules is reference single nucleotide polymorphism (SNP) (RS) numbers or genomic coordinates (GRCh37) of the query variants. When RS numbers are provided as input, an indexed database of dbSNP version 151 (Database of Single Nucleotide Polymorphisms [DBSNP], 2007) is used to match query RS numbers with the genomic coordinates (GRCh37) of the SNPs of interest. The *LDlinkR* R package includes seven main modules (LDhap, LDmatrix, LDpair, LDpop, LDproxy, SNPchip, and SNPclip) that can be used to carry out a variety of LD-based calculations (Figure 1).

**FIGURE 2:**
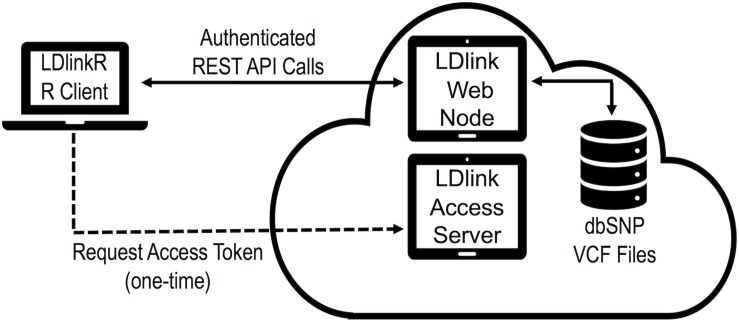
Diagram of *LDlinkR* API call to the LDlink web server.

The design goal of *LDlinkR* is to easily perform LD calculations utilizing the R client interface (Figure 2). The *LDlinkR* package provides multiple annotated functions to easily generate data in similar formats as produced by the main LDlink web modules and store these data locally for further analysis in R. Function names in *LDlinkR* correspond to the names of popular LDlink modules they are designed to generate output for. Available functions include LDhap, LDmatrix, LDpair, LDpop, LDproxy, LDproxy_batch, SNPchip, and SNPclip. We currently do not have an implementation for LDassoc in *LDlinkR* due to challenges in managing large datasets from GWAS. Additionally, *LDlinkR* provides two utility functions. The first, list_pop, provides users with available super-populations and sub-populations along with their corresponding three-letter designation. The second, list_chip, produces a list of available SNPchip arrays for querying by the SNPchip module. All functions require an RS number/genomic coordinate or a list of RS numbers/genomic coordinates as an input argument. Selection of 1000G populations is necessary if the population of interest is other than the default provided. In general, output returned by *LDlinkR* is formatted to match the file format downloaded from the LDlink web site.

Installation of the release version of *LDlinkR* can be installed from CRAN with:

>install.packages⁢(`⁢`⁢LDlinkR⁢”)

The development version of the *LDlinkR* package can be installed from the GitHub repository by using the R package *devtools* with:

>devtools::install_github(``CBIIT/LDlinkR”)

Following installation, attach the *LDlinkR* package:

>library⁢(LDlinkR)

Additionally, an access token is required for each *LDlinkR* call to manage access and usage to our cloud-based servers. Users can register for access tokens at https://ldlink.nci.nih.gov/?tab=apiaccess. Tokens are automatically generated and emailed to users. Questions about access tokens, installation issues, fixes or help may be posted by opening an issue on the GitHub repository^2^ or by sending an email to NCILDlinkWebAdmin@mail.nih.gov.

The performance of each *LDlinkR* function was measured using the R package *bench* v.1.1.1 (RStudio Team, 2016; Hester, 2020; Table 1). Execution time and memory allocation were analyzed on a MacBook Pro (2.6 GHz Intel Core i7, 16 GB RAM) running macOS Mojave (v. 10.14.6) on a gigabit ethernet network connected to the internet by high-speed connection. However, performance will vary depending on a variety of factors such as CPU speed, memory, internet connection speed and user input (number of query variants and populations, and number of dbSNP variants in the region queried).

**TABLE 1:** Computational performance for a typical execution of each *LDlinkR* function.

**LDlinkR function**	**Number of variants queried**	**Min. execution time (s)**	**Median execution time (s)**	**Estimated number of executions per second**	**Memory allocated by R (kb)**	**Total number of iterations**
LDhap	3	0.63	0.70	1.41	135	10
LDmatrix	3	0.93	1.09	0.63	118	10
LDpair	2	0.80	0.94	1.07	202	9
LDpop	2	0.72	0.77	1.29	191	10
LDproxy	1	9.10	9.91	0.09	1089	9
LDproxy_batch	2	18.37	20.82	0.05	1633	10
SNPchip	3	0.25	0.28	3.57	194	10
SNPclip	3	0.34	0.39	2.44	119	10

*LDlinkR* was written running macOS Sierra version 10.12.6 with the R Studio version 1.0.153 integrated development environment (IDE) (RStudio Team, 2016) using R version 3.5.2 (R Core Team, 2018). The *LDlinkR* library was tested on a variety of operating systems and R versions to ensure cross-platform compatibility. LDlink modules are written in Python 2.7 and run on a cloud-based server (Machiela and Chanock, 2015). Tabix version 0.2.5 is used to access phased genotypes of query variants from indexed VCF files (Li et al., 2011).

## Usage and Examples

We have included examples to demonstrate the query structure and utility of *LDlinkR*. The first example retrieves proxy and putatively functional variants for a query variant using the *LDlinkR* LDproxy function. Typical input is a single RS number or genomic coordinate (GRCh37) and a population or populations of interest. The returned output is a data frame of proxy variants −/+500 Kb from the query variant with a pairwise *R*^2^ value greater than 0.01. In this example, we are interested in investigating LD of variant rs2887399 which has been identified by a GWAS designed to find genomic regions associated with risk for the mosaic loss of chromosome Y (mLOY) (Zhou et al., 2016). Retrieving proxy variants for the query variant rs2887399 in the CEU population can be carried out by executing the simple command:

>df_proxies<-LDproxy(``rs2887399”,pop=``CEU”,

r2d=``r2”,token=``YourTokenHere123”)

This code returns a data frame of 1,454 proxy variants with variant details returned (e.g., RS_Number, Coord, Alleles, MAF, Distance, Dprime, R2, Correlated_Alleles, RegulomeDB, and Function). If a researcher desires to investigate more than one query variant, the *LDlinkR* LDproxy_batch function accepts a list of query variants and generates sequential API calls for each variant.

Next, we will use base R code to subset the list of proxy variants stored in the “RS_Number” column of the df_proxies variable using an *R*^2^ threshold greater than or equal to 0.8:

>df_proxies_sub<-as.character(with(df_proxies,

subset(RS_Number,R2≥0.80)))

The result is a character vector of 13 variants which will then be passed to the LDhap function to calculate population-specific haplotype frequencies of all haplotypes observed for this subset of variants in the available European (EUR) sub-populations:

>LDhap(snps=df_proxies_sub,pop=``EUR”,token

=``YourTokenHere123”)

LDhap returns a data frame of observed haplotypes with frequencies greater than one percent and ordered by observed frequency in the selected query sub-populations.

The next example utilizes the *LDlinkR* LDpair function to investigate the correlated alleles for the query variant, rs2887399, from the example above with one of its proxy variants, rs1957940, in all available EUR sub-populations. In addition to a pair of variants and populations of interest, an optional argument will provide output data formatted in either table or text format. For this example, the following function call will return the desired result in table format:

>LDpair(var1=``rs2887399”,var2=``rs1957940”,

pop=`⁢`⁢EUR⁢”,token=`⁢`⁢YourTokenHere123⁢”,

output=``table”)

In order to investigate allele frequencies and LD patterns across all available 1000G human populations of variants rs2887399 and rs1957940:

>LDpop(var1=``rs2887399”,var2=

``rs1957940”,pop=``ALL”,token=``YourTokenHere123”)

A data frame is generated showing allele frequency and LD values for the query variants and selected 1000G human populations.

Extended examples and descriptions of each *LDlinkR* function are presented in the Supplementary Material and can be viewed at the *LDlinkR* GitHub repository (see text footnote 2).

## Conclusion

Here we introduce the *LDlinkR* package which provides a native R environment for calculation of expansive lists of LD statistics. While the web-based LDlink is a widely used suite of applications, limitations in submitting multiple queries for long lists of variants and downloading these results have led to the development and advanced features of *LDlinkR*. Our expectation is that the *LDlinkR* package will aid the scientific community in performing large queries and accelerate biomedical research that relies on accurate and population-specific measures of LD.

## Data Availability Statement

Publicly available datasets were analyzed in this study. This data can be found here: ftp://ftp.1000genomes.ebi.ac.uk/vol1/ftp/release/20130502/.

## Author Contributions

MM and TM conceived of the project, developed the R package, and wrote the manuscript and documentation. MM conceived and developed the LDlink suite of web-applications in Python and supervised the project. SC provided the financial support and reviewed the final manuscript.

## Conflict of Interest

The authors declare that the research was conducted in the absence of any commercial or financial relationships that could be construed as a potential conflict of interest.

## Footnotes

1 https://ldlink.nci.nih.gov

2 https://github.com/CBIIT/LDlinkR

## Supplementary Material

FILE S1 Extended examples and descriptions of *LDlinkR* functions.
