# Supplementary material for: LDlinkR: An R Package for Rapidly Calculating Linkage Disequilibrium Statistics in Diverse Populations
Source: Front Genet. 2020 Feb 28;11:157. doi: 10.3389/fgene.2020.00157 (PMC7059597; doi:10.3389/fgene.2020.00157)
Supplement: FILE S1 — Extended examples and descriptions of LDlinkR functions. [file Data_Sheet_1.PDF]

LDlinkR: An R Package for Rapidly Calculating Linkage Disequilibrium Statistics in Diverse Populations

Timothy A. Myers, Stephen J. Chanock and Mitchell J. Machiela

24 Jan 2020

Contents

Description 1
Installation 2
Personal Access Token - Required 2
Functions 3
LDhap 3
LDmatrix 4
LDpair 6
LDpop 7
LDproxy 8
LDproxy_batch 9
SNPchip 10
SNPclip 12
Utilities 13
list_chips 13
list_pop 15
FAQs (Frequently Asked Questions) 16
Session Information 17

Description

LDlink is an interactive and powerful suite of web-based tools for querying germline variants in human population groups of interest to generate interactive tables and plots.

LDlinkR is an R package developed to query and download results generated by LDlink web-based applications from the R console. *LDlinkR* accelerates genomic research by providing efficient and user-friendly functions to programmatically interrogate pairwise linkage disequilibrium from large lists of variants.

Installation

- The release version of *LDlinkR* can be installed from CRAN:

```
install.packages("LDlinkR")
```

- The development version of the *LDlinkR* package can be installed from the GitHub repository by using the *devtools* package:

```
devtools::install_github("CBIIT/LDlinkR")
```

LDlinkR depends on the following packages:

- *utils*, version 3.4.2 or later
- *httr*, version 1.4.0 or later

Following installation, attach the *LDlinkR* package with:

```
library(LDlinkR)
```

Personal Access Token - Required

In order to access the LDlink API via *LDlinkR*, we use a personal access token. This is a common convention followed by many APIs and emulates the more familiar HTTPS username/password or SSH keys.

You will need to:

- Make a one-time request for your personal access token from a web browser at https://ldlink.nci.nih.gov/?tab=apiaccess.
- Once registered, your personal access token will be emailed to you. It is a string of 12 random letters and numbers.
- Provide your token as an argument when using *LDlinkR*. See example below:

```
LDhap(snps = c("rs3", "rs4", "rs148890987"),
      pop = "YRI",
      token = "YourTokenHere123")
```

Optional: However, the best security practice is to store your personal access token as an envionment variable where *LDlinkR* can find it and use it on your behalf but where it will not be accidentally shared with the public. **Note:** Modifying R startup files (such as the `.Renviron`) is for the advanced R user only. Modification of these files in the wrong way could cause problems. Please proceed cautiously. Step-by-step instructions follow:

After retrieving your personal access token from your email, put your token in your `.Renviron` file. `.Renviron` is a hidden file that lives in your home directory. The easiest way to both find and edit the `.Renviron` file is with a function from the *usethis* package. From the R console, do:

```
usethis::edit_r_environ()
```

Your `.Renviron` file should open in your editor. Add a line that looks like this:

```
LDLINK_TOKEN=YourTokenHere123
```

Important, ensure you put a line break at the end by hitting the *enter/return* key.

Save and close the `.Renviron` file. Restart R, as environment variables are only loaded from `.Renviron` at the start of a new R session. Now, check to see that your token is available by entering:

```
Sys.getenv("LDLINK_TOKEN")
```

```
## [1] "YourTokenHere123"
```

You should see your personal access token print to the screen, as shown above. Now, *LDlinkR* function calls that use

```
Sys.getenv("LDLINK_TOKEN")
```

for the `token` argment in *LDlinkR* function calls will use your personal access token in a private and secure way. This method will be used in the extended examples that follow.

Functions

LDhap

Function

```
LDhap(snps, pop="CEU", token=NULL, file = FALSE)
```

Calculates population specific haplotype frequencies of all haplotypes observed for a list of query variants. Input is a list of variant RS numbers (concatenated list) and a population group.

Arguments

- `snps`, a list of between 1 - 30 variants, using an rsID or chromosome coordinate (e.g. "chr7:24966446")
- `pop`, a 1000 Genomes Project population, uses three letter population code, (e.g. YRI or CEU), multiple allowed, default = "CEU"
- `token`, LDlink provided user access token is required, default = NULL
- `file`, optional character string naming a path and file for saving results. If file = FALSE, no file will be generated, default = FALSE

```
LDhap(snps = c("rs3", "rs4", "rs148890987"),
      pop = "CEU",
      token = Sys.getenv("LDLINK_TOKEN")
     )
```

Usage: Multiple query variants, single population

```
##   rs148890987 rs3 rs4 Count Frequency
## 1           C   C   A   176    0.8889
## 2           T   T   G    11    0.0556
## 3           T   C   A     7    0.0354
## 4           C   T   G     4    0.0202
```

```r
LDhap(snps = c("rs3", "rs4", "rs148890987"),
      pop = c("YRI", "CEU"),
      token = Sys.getenv("LDLINK_TOKEN")
     )
```

Usage: Multiple query variants, multiple populations

```
##   rs148890987 rs3 rs4 Count Frequency
## 1           C   C   A   355    0.8575
## 2           C   T   G    41     0.099
## 3           T   T   G    11    0.0266
## 4           T   C   A     7    0.0169
```

Output is a table of alleles, haplotype count and haplotype frequencies.

LDmatrix

Function

```
LDmatrix(snps, pop = "CEU", r2d = "r2", token = NULL, file = FALSE)
```

Generates a data frame of pairwise linkage disequilibirum statistics. Input is a list of between 2 to 1000 variants. Desired output can be based on estimates of R^2 or D'.

Arguments

- `snps`, list of between 2 - 1,000 variants, using an rsID or chromosome coordinate (GRCh37/hg19) (e.g. "chr7:24966446")
- `pop`, a 1000 Genomes Project population, uses three letter population code, (e.g. YRI or CEU), multiple allowed, default = "CEU"
- `r2d`, use either "r2" for pairwise R^2 statistics or "d" for pairwise D' statistics
- `token`, LDlink provided user access token is required, default = NULL
- `file`, optional character string naming a path and file for saving results. If file = FALSE, no file will be generated, default = FALSE

```r
LDmatrix(snps = c("rs496202", "rs11147477", "rs201578600"),
         pop = "YRI", r2d = "r2",
         token = Sys.getenv("LDLINK_TOKEN")
        )
```

Usage: Multiple query variants, single population, R^2

```
##     RS_number rs496202 rs201578600 rs11147477
## 1    rs496202    1.000       0.660      0.504
## 2 rs201578600    0.660       1.000      0.786
## 3  rs11147477    0.504       0.786      1.000
```

```
LDmatrix(snps = c("chr13:32444611", "rs11147477", "rs201578600"),
         pop = c("YRI", "CEU"), r2d = "d",
         token = Sys.getenv("LDLINK_TOKEN")
        )
```

Usage: Multiple query variants (rsID & genomic coordinates), multiple populations, D'

```
##     RS_number rs496202 rs201578600 rs11147477
## 1    rs496202    1.000       0.973      0.738
## 2 rs201578600    0.973       1.000      0.971
## 3  rs11147477    0.738       0.971      1.000
```

```
my_variants <- read.table("variant_list.txt")
my_variants
```

Usage: Multiple query variants read from text file, multiple populations, D'

```
##           V1
## 1      rs456
## 2      rs114
## 3      rs127
## 4  rs7805287
## 5 rs60676332
## 6 rs10239961
```

Then, call *LDmatrix* with:

```
LDmatrix(snps = my_variants[,1],
         pop = c("YRI", "CEU"), r2d = "d",
         token = Sys.getenv("LDLINK_TOKEN")
        )
```

```
##    RS_number rs60676332 rs7805287 rs127 rs456 rs10239961 rs114
## 1 rs60676332      1.000     0.094 0.180 0.151      0.363 0.148
## 2  rs7805287      0.094     1.000 0.818 0.789      0.464 0.710
## 3      rs127      0.180     0.818 1.000 0.929      0.912 0.886
## 4      rs456      0.151     0.789 0.929 1.000      1.000 0.963
## 5 rs10239961      0.363     0.464 0.912 1.000      1.000 0.459
## 6      rs114      0.148     0.710 0.886 0.963      0.459 1.000
```

Output is a table with rows and columns equal to the number of query variants and pairwise linkage disequilibrium statistics.

LDpair

Function

```
LDpair(var1, var2, pop = "CEU", token = NULL, output = "table", file = FALSE)
```

Investigates potentially correlated alleles for a pair of variants. Input is two query variants and a 1000 Genomes Project reference population(s) of interest.

Arguments

- `var1`, the first RS number (rsID) or genomic coordinate (GRCh37/hg19) (e.g. "chr7:24966446"), must match a bi-allelic variant
- `var2`, the second RS number or genomic coordinate, as above, must match a bi-allelic variant
- `pop`, a 1000 Genomes Project reference population, uses three letter population code, (e.g. YRI or CEU), multiple allowed, default = "CEU"
- `token`, LDlink provided user access token is required, default = NULL
- `output`, two output format options are available, "text", which displays a two-by-two matrix displaying haplotype counts and allele frequencies along with other statistics, or "table", which displays the same data in rows and columns, default = "table"
- `file`, optional character string naming a path and file for saving results. If file = FALSE, no file will be generated, default = FALSE

```r
LDpair(var1 = "rs496202",
       var2 = "rs11147477",
       pop = "YRI",
       token = Sys.getenv("LDLINK_TOKEN"),
       output = "text"
      )
```

Usage: With `output` argument set to "text"

```
## Query SNPs:
## rs496202 (chr13:32444611)
## rs11147477 (chr13:32509120)
##
## YRI Haplotypes:
##                 rs11147477
##                 C       T
##               -----------------
##            C | 11    | 26    | 37    (0.171)
## rs496202     -----------------
##            G | 173   | 6     | 179   (0.829)
##               -----------------
```

```
##                184     32      216
##              (0.852) (0.148)
##
##          G_C: 173 (0.801)
##          C_T: 26 (0.12)
##          C_C: 11 (0.051)
##          G_T: 6 (0.028)
##
##          D': 0.7737
##          R2: 0.5037
##      Chi-sq: 108.8005
##     p-value: <0.0001
##
## rs496202(C) allele is correlated with rs11147477(T) allele
## rs496202(G) allele is correlated with rs11147477(C) allele
```

```
LDpair(var1 = "rs496202",
       var2 = "rs11147477",
       pop = "YRI",
       token = Sys.getenv("LDLINK_TOKEN")
      )
```

Usage: With no output argument option specified, using default "table"

```
##       var1       var2 pops       var1_pos       var2_pos var1_a1 var1_a2
## 1 rs496202 rs11147477  YRI chr13:32444611 chr13:32509120       C       G
##   var1_a1_freq var1_a2_freq var2_a1 var2_a2 var2_a1_freq var2_a2_freq
## 1        0.171        0.829       C       T        0.852        0.148
##   d_prime     r2    chisq p_val
## 1  0.7737 0.5037 108.8005 1e-04
##                                               corr_alleles
## 1 rs496202(C)-rs11147477(T), rs496202(G)-rs11147477(C)
```

Output of the `output` argument "text" option is a two-by-two contingency table displaying haplotype counts and allele frequencies of the two query variants. Also displayed are calculated metrics of linkage disequilibrium including: D prime (D'), R square (R^2), and goodness-of-fit (Chi-square and p-value). Goodness-of-fit tests for deviations of expected haplotype frequencies based on allele frequencies. Correlated alleles are reported if linkage disequilibrium is present ($R^2 > 0.1$). If linkage equilibrium, no alleles are reported.

Output from the `output` argument "table" option converts the data from the two-by-two contingency table into a data frame.

LDpop

Function

```
LDpop(var1, var2, pop = "CEU", r2d = "r2", token = NULL, file = FALSE)
```

Investigates allele frequencies and linkage disequilibrium patterns across 1000G populations.

Arguments

- `var1`, the first RS number (rsID) or genomic coordinate (GRCh37/hg19) (e.g. "chr7:24966446"), must match a bi-allelic variant
- `var2`, the second RS number or genomic coordinate, as above, must match a bi-allelic variant
- `pop`, a 1000 Genomes Project reference population, uses three letter population code, (e.g. YRI or CEU), multiple allowed, default = "CEU"
- `r2d`, use "r2" if desired output is based on estimated R^2 or "d" if D'
- `token`, LDlink provided user access token is required, default = NULL
- `file`, optional character string naming a path and file for saving results. If file = FALSE, no file will be generated, default = FALSE

```
LDpop(var1 = "rs496202",
      var2 = "rs11147477",
      pop = "YRI",
      r2d = "r2",
      token = Sys.getenv("LDLINK_TOKEN")
     )
```

Usage

```
##   Population   N rs496202_Allele_Freq rs11147477_Allele_Freq     R2     D'
## 1        YRI 108 G: 82.87%, C: 17.13%   C: 85.19%, T: 14.81% 0.5037 0.7737
```

LDproxy

Function

```
LDproxy(snp, pop = "CEU", r2d = "r2", token = NULL, file = FALSE)
```

Explore proxy and putatively functional variants for a single query variant. Input is a single RS number and a population group. Depending on the number of query populations, this function could take some time to run.

Arguments

- `snp`, an RS number (rsID) or chromosome coordinate (GRCh37/hg19) (e.g. "chr7:24966446"), one per query, RS number must match a bi-allelic variant
- `pop`, a 1000 Genomes Project reference population, uses three letter population code, (e.g. YRI or CEU), multiple allowed, default = "CEU"
- `r2d`, use "r2" if desired output is based on estimated R^2 or "d" if D'
- `token`, LDlink provided user access token is required, default = NULL
- `file`, optional character string naming a path and file for saving results. If file = FALSE, no file will be generated, default = FALSE

```r
my_proxies <- LDproxy(snp = "rs456",
                      pop = "YRI",
                      r2d = "r2",
                      token = Sys.getenv("LDLINK_TOKEN")
                     )
```

Usage: single reference population Output is a data frame stored in the variable `my_proxies` with 2455 rows and 10 columns with data.

```r
head(my_proxies)
```

```
##    RS_Number         Coord Alleles    MAF Distance Dprime     R2
## 1      rs456 chr7:24962419   (G/C) 0.1944        0      1 1.0000
## 2      rs457 chr7:24962426   (T/C) 0.1944        7      1 1.0000
## 3 rs28475742 chr7:24964633   (G/T) 0.1944     2214      1 1.0000
## 4      rs123 chr7:24966446   (C/A) 0.1944     4027      1 1.0000
## 5      rs125 chr7:24959703   (C/T) 0.2037    -2716      1 0.9436
## 6      rs128 chr7:24958977   (C/T) 0.2037    -3442      1 0.9436
##   Correlated_Alleles RegulomeDB Function
## 1            G=G,C=C          5     <NA>
## 2            G=T,C=C          5     <NA>
## 3            G=G,C=T          4     <NA>
## 4            G=C,C=A         1f     <NA>
## 5            G=C,C=T          5     <NA>
## 6            G=C,C=T          7     <NA>
```

Includes information on all variants -/+ 500 Kb of the query variant with a pairwise R^2 value greater than 0.01.

LDproxy_batch

Function

```
LDproxy_batch(snp, pop = "CEU", r2d = "r2", token = NULL, append = FALSE)
```

Query LDproxy using a list of query variants. *LDproxy_batch* will make sequential queries, one query per variant. Concurrent queries are not permitted by the LDlink API. Ouptput is saved as text file(s) to the current working directory. Depending on the number of query variants and reference populations selected, this function could time some time to run.

Arguments

- `snp`, a character string or data frame listing RS numbers (rsID) or chromosome coordinates (GRCh37/hg19) (e.g. “chr7:24966446”), one per line.
- `pop`, a 1000 Genomes Project reference population, uses three letter population code, (e.g. YRI or CEU), multiple allowed, default = “CEU”
- `r2d`, use “r2” if desired output is based on estimated R^2 or “d” if D’

- `token`, LDlink provided user access token is required, default = NULL
- `append`, a logical, if TRUE, output for eqch query variant is appended to a single text file and saved to the current working directory. If FALSE, output for each query variant is saved in its own text file with the query variant as the filename. Default value is FALSE.

Usage: multiple variants, default `pop` and `r2d` The list of query variants passed to *LDproxy_batch* can be stored as a character string.

```
LDproxy_batch(snp = c("rs456", "rs114", "rs127"),
              token = Sys.getenv("LDLINK_TOKEN")
             )
```

Or, a longer list of variants can be read into a data frame from a text file and passed into *LDproxy_batch*. The list should be in a simple text file, one query variant per line. For example:

```
my_variants <- read.table("variant_list.txt")
my_variants
```

```
##           V1
## 1      rs456
## 2      rs114
## 3      rs127
## 4  rs7805287
## 5 rs60676332
## 6 rs10239961
```

Then, call *LDproxy_batch* with:

```
LDproxy_batch(snp = my_variants,
              token = Sys.getenv("LDLINK_TOKEN")
             )
```

Output not displayed. All output from *LDproxy_batch* is saved to a text file(s) in the current working directory.

SNPchip

Function

```
SNPchip(snps, chip = "ALL", token = NULL, file = FALSE)
```

Used to find commercial genotyping chip arrays for variants. Input is a list of between 1 - 5000 variants (one per line) and desired commercial chip arrays to search. Input variants do not need to be on the same chromosome.

Arguments

- `snps`, between 1 - 5,000 variants, using an rsID or chromosome coordinate (e.g. "chr7:24966446")
- `chip`, chip or arrays, platform code(s) for a SNP chip array, ALL_Illumina, ALL_Affy or ALL, default=ALL, use the `list_chips` utility (see below) to lookup available commercial SNP chip arrays and their codes.
- `token`, LDlink provided user access token is required is required, default = NULL
- `file`, optional character string naming a path and file for saving results. If file = FALSE, no file will be generated, default = FALSE

```
SNPchip(snps = c("rs3", "rs4", "rs148890987"),
        chip = "ALL",
        token = Sys.getenv("LDLINK_TOKEN")
       )
```

Usage: Multiple variants, search "ALL" available chip arrays

```
## WARNING: The following RS number did not have any platforms found: rs148890987, rs3.

##     RS_Number Position_GRCh37 A_SNP5.0 A_CHB2 A_250S A_SNP6.0
## 1 rs148890987  chr13:32403784        0      0      0        0
## 2         rs3  chr13:32446842        0      0      0        0
## 3         rs4  chr13:32447222        1      1      1        1
```

```
SNPchip(snps = c("rs3", "rs4", "rs148890987"),
        chip = c("A_SNP5.0", "A_CHB2"),
        token = Sys.getenv("LDLINK_TOKEN")
       )
```

Usage: Multiple variants, search two Affymetrix arrays

```
## WARNING: The following RS number did not have any platforms found: rs148890987, rs3.

##     RS_Number Position_GRCh37 A_SNP5.0 A_CHB2
## 1 rs148890987  chr13:32403784        0      0
## 2         rs3  chr13:32446842        0      0
## 3         rs4  chr13:32447222        1      1
```

```
SNPchip(snps = c("rs3", "rs4", "rs148890987"),
        chip = "ALL_Affy",
        token = Sys.getenv("LDLINK_TOKEN")
       )
```

Usage: Multiple variants, search all available Affymetrix arrays using, "ALL_Affy"

```
## WARNING: The following RS number did not have any platforms found: rs148890987, rs3.
```

```
##     RS_Number Position_GRCh37 A_SNP5.0 A_CHB2 A_250S A_SNP6.0
## 1 rs148890987  chr13:32403784        0      0      0        0
## 2         rs3  chr13:32446842        0      0      0        0
## 3         rs4  chr13:32447222        1      1      1        1
```

Output is a data frame of query variant rows (RS number), genomic coordinate (GRCh37) and genotyping chip array columns. The presence of a "1" designates the variant is present on the respective commercial genotyping array and a "0" indicates that it is not present on the genotyping array.

SNPclip

Function

```
SNPclip(snps, pop = "CEU", r2_threshold = "0.1", maf_threshold = "0.01", token = NULL, file = FALSE)
```

Prune a list of variants by linkage disequilibrium. Input is a list of variant RS numbers (one per line) and a population group.

Arguments

- `snps`, a list of between 1 - 5,000 variants, using an RS number (rsID) or chromosome coordinate (GRCh37) (e.g. "chr7:24966446"). All input variants must be on the same chromosome and match a bi-allelic variant.
- `pop`, a 1000 Genomes Project reference population, uses three letter population code, (e.g. YRI or CEU), multiple allowed, default = "CEU"
- `r2_threshold`, Used to set the R^2 threshold for LD pruning. One of each pair of variants with a R^2 greater than the threshold is removed. Value needs to be in the range 0 to 1. Default value is 0.1.
- `maf_threshold`, Used to set minor allele frequency (MAF) threshold for LD pruning. Variants with a MAF less than or equal to the threshold are removed. Value needs to be in the range 0 to 1. Default value is 0.01.
- `token`, LDlink provided user access token is required is required, default = NULL
- `file`, optional character string naming a path and file for saving results. If file = FALSE, no file will be generated, default = FALSE

```r
SNPclip(snps =  c("rs3", "rs4", "rs148890987", "rs115955931"),
        pop = "YRI",
        r2_threshold =  "0.1",
        maf_threshold = "0.01",
        token = Sys.getenv("LDLINK_TOKEN")
       )
```

Usage: Multiple Variants

```
##     RS_Number       Position          Alleles
## 1         rs3 chr13:32446842 C=0.829, T=0.171
## 2         rs4 chr13:32447222 A=0.829, G=0.171
## 3 rs148890987 chr13:32403784     C=1.0, T=0.0
## 4 rs115955931 chr13:32130008 G=0.954, A=0.046
##                                               Details
## 1                                       Variant kept.
## 2 Variant in LD with rs3 (R2=1.0), variant removed.
## 3             Variant MAF is 0.0, variant removed.
## 4                                       Variant kept.
```

The output table provides details including query varaint RS number, genomic position, alleles, and and details about whether the variant was kept or removed.

Utilities

list_chips

Function

```
list_chips()
```

Provides a data frame listing the names and abbreviation codes for available commerical SNP Chip Arrays from Illumina and Affymetrix.

```
list_chips()
```

Usage

```
##         chip_code                          chip_name
## 1       A_Exome1A            Affymetrix Axiom Exome 1A
## 2      A_Exome319           Affymetrix Axiom Exome 319
## 3           A_AFR              Affymetrix Axiom GW AFR
## 4           A_ASI              Affymetrix Axiom GW ASI
## 5          A_CHB2             Affymetrix Axiom GW CHB2
## 6           A_EAS              Affymetrix Axiom GW EAS
## 7           A_EUR              Affymetrix Axiom GW EUR
## 8            A_Hu               Affymetrix Axiom GW Hu
## 9        A_Hu-CHB           Affymetrix Axiom GW Hu-CHB
## 10          A_LAT              Affymetrix Axiom GW LAT
## 11      A_DMETplus               Affymetrix DMET Plus
## 12          A_10X        Affymetrix Mapping 10K Xba142
## 13         A_250N         Affymetrix Mapping 250K Nsp
## 14         A_250S         Affymetrix Mapping 250K Sty
## 15          A_50H       Affymetrix Mapping 50K Hind240
## 16          A_50X        Affymetrix Mapping 50K Xba240
## 17         A_Onco                 Affymetrix OncoScan
```

```
## 18      A_OncoCNV                 Affymetrix OncoScan CNV
## 19       A_SNP5.0                      Affymetrix SNP 5.0
## 20       A_SNP6.0                      Affymetrix SNP 6.0
## 21  I_CardioMetab            Illumina Cardio-MetaboChip
## 22          I_1M-D                Illumina Human1M-Duov3
## 23            I_1M                    Illumina Human1Mv1
## 24         I_610-Q              Illumina Human610-Quadv1
## 25        I_660W-Q             Illumina Human660W-Quadv1
## 26        I_CNV-12                  Illumina HumanCNV-12
## 27      I_CNV370-D            Illumina HumanCNV370-Duov1
## 28      I_CNV370-Q           Illumina HumanCNV370-Quadv3
## 29       I_Core-12                Illumina HumanCore-12v1
## 30    I_CoreE-12v1          Illumina HumanCoreExome-12v1
## 31  I_CoreE-12v1.1        Illumina HumanCoreExome-12v1.1
## 32    I_CoreE-24v1          Illumina HumanCoreExome-24v1
## 33  I_CoreE-24v1.1        Illumina HumanCoreExome-24v1.1
## 34           I_CVD                    Illumina HumanCVDv1
## 35      I_Cyto-12v2           Illumina HumanCytoSNP-12v2
## 36    I_Cyto-12v2.1         Illumina HumanCytoSNP-12v2.1
## 37  I_Cyto-12v2.1f    Illumina HumanCytoSNP-12v2.1 FFPE
## 38      I_Exome-12           Illumina HumanExome-12v1.1
## 39      I_Exon510S             Illumina HumanExon510Sv1
## 40          I_240S                 Illumina HumanHap240S
## 41        I_300-D            Illumina HumanHap300-Duov2
## 42           I_300                Illumina HumanHap300v1
## 43         I_550v1                Illumina HumanHap550v1
## 44         I_550v3                Illumina HumanHap550v3
## 45          I_650Y               Illumina HumanHap650Yv3
## 46   I_Immuno-24v1             Illumina HumanImmuno-24v1
## 47   I_Immuno-24v2             Illumina HumanImmuno-24v2
## 48     I_Linkage-12              Illumina HumanLinkage-12
## 49     I_Linkage-24              Illumina HumanLinkage-24
## 50         I_NS-12                   Illumina HumanNS-12
## 51          I_O1-Q            Illumina HumanOmni1-Quadv1
## 52         I_O1S-8              Illumina HumanOmni1S-8v1
## 53        I_O2.5-4             Illumina HumanOmni2.5-4v1
## 54        I_O2.5-8           Illumina HumanOmni2.5-8v1.2
## 55     I_O2.5E-8v1        Illumina HumanOmni2.5Exome-8v1
## 56   I_O2.5E-8v1.1      Illumina HumanOmni2.5Exome-8v1.1
## 57   I_O2.5E-8v1.2      Illumina HumanOmni2.5Exome-8v1.2
## 58       I_O2.5S-8            Illumina HumanOmni2.5S-8v1
## 59          I_O5-4               Illumina HumanOmni5-4v1
## 60         I_O5E-4          Illumina HumanOmni5Exome-4v1
## 61         I_OE-12        Illumina HumanOmniExpress-12v1
## 62        I_OE-12f   Illumina HumanOmniExpress-12v1 FFPE
## 63         I_OE-24        Illumina HumanOmniExpress-24v1
## 64       I_OEE-8v1     Illumina HumanOmniExpressExome-8v1
## 65     I_OEE-8v1.1  Illumina HumanOmniExpressExome-8v1.1
## 66     I_OEE-8v1.2  Illumina HumanOmniExpressExome-8v1.2
## 67     I_OEE-8v1.3  Illumina HumanOmniExpressExome-8v1.3
## 68       I_OZH-8v1        Illumina HumanOmniZhongHua-8v1
## 69     I_OZH-8v1.1      Illumina HumanOmniZhongHua-8v1.1
## 70     I_OZH-8v1.2      Illumina HumanOmniZhongHua-8v1.2
## 71      I_Cyto850         Illumina Infinium CytoSNP-850K
```

```
## 72          I_100           Illumina Infinium Human100kv1
## 73  I_ME-Global-8 Illumina Infinium Multi-Ethnic Global-8
## 74    I_OncoArray        Illumina Infinium OncoArray-500K
## 75    I_Psyc-24v1       Illumina Infinium PsychArray-24v1
## 76  I_Psyc-24v1.1     Illumina Infinium PsychArray-24v1.1
```

---

list_pop

Function

`list_pop()`

Provides a data frame listing the available reference populations from the 1000 Genomes Project, continetal or super-populations (e.g. European, African, Admixed American) and sub-populations (e.g Finnish, Gambian, Peruvian)

```r
list_pop()
```

Usage

```
##    pop_code super_pop_code                                pop_name
## 1       ALL            ALL                         ALL POPULATIONS
## 2       AFR            AFR                                 AFRICAN
## 3       YRI            AFR                 Yoruba in Ibadan, Nigera
## 4       LWK            AFR                   Luhya in Webuye, Kenya
## 5       GWD            AFR                Gambian in Western Gambia
## 6       MSL            AFR                    Mende in Sierra Leone
## 7       ESN            AFR                           Esan in Nigera
## 8       ASW            AFR   Americans of African Ancestry in SW USA
## 9       ACB            AFR          African Carribbeans in Barbados
## 10      AMR            AMR                       AD MIXED AMERICAN
## 11      MXL            AMR    Mexican Ancestry from Los Angeles, USA
## 12      PUR            AMR           Puerto Ricans from Puerto Rico
## 13      CLM            AMR         Colombians from Medellin, Colombia
## 14      PEL            AMR                Peruvians from Lima, Peru
## 15      EAS            EAS                              EAST ASIAN
## 16      CHB            EAS              Han Chinese in Bejing, China
## 17      JPT            EAS                  Japanese in Tokyo, Japan
## 18      CHS            EAS                      Southern Han Chinese
## 19      CDX            EAS        Chinese Dai in Xishuangbanna, China
## 20      KHV            EAS          Kinh in Ho Chi Minh City, Vietnam
## 21      EUR            EUR                                  EUROPEAN
## 22      CEU            EUR Utah Residents from North and West Europe
## 23      TSI            EUR                         Toscani in Italia
## 24      FIN            EUR                        Finnish in Finland
## 25      GBR            EUR            British in England and Scotland
## 26      IBS            EUR              Iberian population in Spain
```

```
## 27      SAS            SAS                               SOUTH ASIAN
## 28      GIH            SAS  Gujarati Indian from Houston, Texas, USA
## 29      PJL            SAS             Punjabi from Lahore, Pakistan
## 30      BEB            SAS                   Bengali from Bangladesh
## 31      STU            SAS              Sri Lankan Tamil from the UK
## 32      ITU            SAS                  Indian Telugu from the UK
```

FAQs (Frequently Asked Questions)

1. What if my access token doesn't work?

   - Please double check that the token was typed accurately. Then, ensure the format of the function call is correct. For example, if your alphanumeric access token is: 123abc456789, then, use it as:

```
df <- LDproxy(snp = "rs456", pop = "YRI", token = "123abc456789")
```

   If you still can not solve the problem, please email us at NCILDlinkWebAdmin@mail.nih.gov.

2. Can I set a threshold or cut-off value for R^2 or D‘ values?

   - No. *LDlinkR* functions do not include 'threshold' as an argument. However, the returned data object can be subset using base R. For example:

```
df <- LDproxy("rs12027135", pop = "CEU",r2d = "r2", token = "YourTokenHere123")
new_df <- subset(df, R2 >= 0.8)
```

3. I need to upload hundreds of variants from a text file into LDmatrix. Why do I get an error with the following code?

```
test <- read.table("variant_list.txt", header = FALSE)
LDmatrix(snps = test, pop = "CEU", r2d = "r2", token = "YourTokenHere123")
```

```
Error in LDmatrix(snps = test, pop = "CEU", r2d = "r2", token = "YourTokenHere123"),  :
Input is between 2 to 1000 variants.
```

- In the above example, 'test' is an object of class data frame and the variants are in the first column. The first column needs to be specified in order to avoid the error. The example below will yield the desired result:

```
test <- read.table("variant_list.txt", header = FALSE)
LDmatrix(snps = test[,1], pop = "CEU", r2d = "r2", token = "YourTokenHere123")
```

```
##    RS_number rs60676332 rs7805287 rs127 rs456 rs10239961 rs114
## 1 rs60676332      1.000     0.008 0.013 0.017      0.286 0.039
## 2  rs7805287      0.008     1.000 0.980 0.882      0.170 0.614
## 3      rs127      0.013     0.980 1.000 0.900      0.167 0.632
## 4      rs456      0.017     0.882 0.900 1.000      0.177 0.722
## 5 rs10239961      0.286     0.170 0.167 0.177      1.000 0.008
## 6      rs114      0.039     0.614 0.632 0.722      0.008 1.000
```

4. What genome build does LDlink use for genomic coordinates?
    - All genomic coordinates are based on GRCh37/hg19.

5. How can I ask for help?
    - If you find that you can't answer a question or solve a problem yourself, you can email us at NCILDlinkWebAdmin@mail.nih.gov or open an issue at the *LDlinkR* GitHub repository (https://github.com/CBIIT/LDlinkR).

Session Information

```r
sessionInfo()
```

```
## R version 3.6.1 (2019-07-05)
## Platform: x86_64-apple-darwin15.6.0 (64-bit)
## Running under: macOS Mojave 10.14.6
##
## Matrix products: default
## BLAS:   /Library/Frameworks/R.framework/Versions/3.6/Resources/lib/libRblas.0.dylib
## LAPACK: /Library/Frameworks/R.framework/Versions/3.6/Resources/lib/libRlapack.dylib
##
## locale:
## [1] en_US.UTF-8/en_US.UTF-8/en_US.UTF-8/C/en_US.UTF-8/en_US.UTF-8
##
## attached base packages:
## [1] stats     graphics  grDevices utils     datasets  methods   base
##
## other attached packages:
## [1] LDlinkR_1.0.1
##
## loaded via a namespace (and not attached):
##  [1] Rcpp_1.0.3      digest_0.6.23   R6_2.4.1        jsonlite_1.6
##  [5] magrittr_1.5    evaluate_0.14   httr_1.4.1      rlang_0.4.2
##  [9] stringi_1.4.3   curl_4.3        rmarkdown_2.0   tools_3.6.1
## [13] stringr_1.4.0   xfun_0.11       yaml_2.2.0      compiler_3.6.1
## [17] htmltools_0.4.0 knitr_1.26
```
